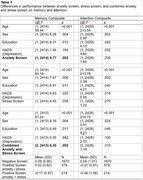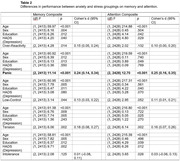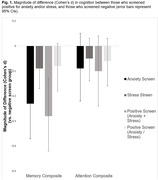# Association between anxiety, stress, and cognition in middle‐aged adults: Exploratory factor analysis across multiple anxiety and stress measures

**DOI:** 10.1002/alz.083960

**Published:** 2025-01-03

**Authors:** Ivana Chan, Yen Ying Lim, Paul Maruff

**Affiliations:** ^1^ Turner Institute for Brain and Mental Health, Melbourne, VIC Australia; ^2^ Turner Institute for Brain and Mental Health, School of Psychological Sciences, Monash University, Melbourne, VIC Australia; ^3^ Cogstate Ltd., Melbourne, VIC Australia

## Abstract

**Background:**

Anxiety and stress have been identified as potential risk factors of cognitive impairment, but research examining this association in midlife has been limited.

**Method:**

The current study examined the associations between anxiety and stress symptoms, and cognition in middle‐aged cognitively unimpaired adults (*n* = 2,463) enrolled in the Healthy Brain Project. Exploratory factor analysis was conducted on items in the Anxiety Subscale of the Hospital Anxiety and Depression Scale, Anxiety and Stress Subscales of the Depression, Anxiety and Stress Scale, and Perceived Stress Scale. Cognition was measured using the Cogstate Brief Battery (CBB).

**Result:**

The solution that resulted in the best fit to the data comprised five factors which explained for 48% of the variance: Over‐Reactivity, Panic, Low‐Control, Tension and Intolerance. Panic‐related symptoms were significantly associated with both poorer attention and memory. Having clinically meaningful anxiety symptoms, or both anxiety and stress symptoms, was associated with poorer memory.

**Conclusion:**

Anxiety and stress symptoms may be linked to a heightened risk of developing cognitive impairment. Specifically, panic‐related symptoms may play a significant role in the relationship between anxiety symptoms and cognition. Limitations include: 1) cross‐sectional nature of the study limited ability to ascertain direction of causality; 2) individuals with major psychiatric conditions were excluded from the study, thus limiting external validity of the current findings; 3) HBP sample has a higher proportion of individuals with a family history of dementia compared to the general population, hence the sample presents with an increased risk of developing dementia and generalizability of our findings may be limited.